# Psychometric Testing of the Chinese Simple Version of the Simulation Learning Effectiveness Inventory: Classical Theory Test and Item Response Theory

**DOI:** 10.3389/fpsyg.2020.00032

**Published:** 2020-02-19

**Authors:** Feifei Huang, Xuan Ye Han, Shiah-Lian Chen, Yu Fang Guo, Anni Wang, Qinghua Zhang

**Affiliations:** ^1^School of Nursing, Fujian Medical University, Fuzhou, China; ^2^Department of Neurosurgery, Second Affiliated Hospital of Harbin Medical University, Harbin, China; ^3^Department of Nursing, National Taichung University of Science and Technology, Taichung, Taiwan; ^4^School of Nursing, Shandong University, Jinan, China; ^5^School of Nursing, Fudan University, Shanghai, China; ^6^School of Nursing, Huzhou University, Huzhou, China

**Keywords:** high-fidelity simulation, nursing education, reliability, item response theory, validity

## Abstract

**Background:**

High-fidelity simulation (HFS) has become a widely used and established pedagogy for teaching clinical nursing skills. Nevertheless, there are few evidence-based instruments that validate the effectiveness of simulation learning in mainland China.

**Methods:**

The Simulation Learning Effectiveness Inventory (SLEI) was adapted and validated for use in this study. Psychometric evaluation, incorporating classical test theory and item response theory (IRT) methods, was performed with 533 third-year undergraduate nursing students who were recruited from May 2017 to July 2018.

**Results:**

The findings of exploratory and confirmatory factor analyses revealed that the simplified Chinese version of the SLEI (SLEI-SC) was composed of six factors, namely, course arrangement, equipment resource, debriefing, clinical ability, problem solving, and confidence, which explained 60.84% of the total variance. The Cronbach’s α, MIIC, marginal reliability, and test–retest reliability values obtained for the total scale were 0.88, 0.38, 0.96, and 0.88, respectively. Furthermore, the difference between the total scores for learning effectiveness pre- and post-course was statistically significant (*t* = 2.59, *p* < 0.05, Cohen’s *d* = 0.60). IRT analysis showed that the SLEI-SC discriminates well between students with high and low levels of learning effectiveness and offers information about a broad range of learning effectiveness measures. The relationship between final course grade and total score on the SLEI-SC was statistically significant (*r* = 0.63, *p* < 0.05).

**Conclusion:**

We demonstrated initial psychometric evidence and support for the 31-item SLEI-SC as a developmentally appropriate instrument for assessing the learning effectiveness of all phases of HFS use with nursing students.

## Introduction

Nursing is a practice-based discipline, and the ultimate goal of nursing education is to help nursing students integrate theoretical knowledge into clinical practice and develop their clinical competence. To achieve clinical competence, the traditional model of school nursing education combined with clinical placements is often considered the gold standard (Pai, 2016). However, nursing students’ direct experience with or opportunities for patient care have been reduced (Kim et al., 2016) due to rapid changes in clinical placements, ethical concerns, and patient safety issues (Yip and Hsiao, 2014). As a useful pedagogical approach, simulation-based education provides nursing students with opportunities to handle various problem-based clinical situations by using patient simulators, such as handling manikins, engaging in role-playing, and practicing on trained persons. Thus, in recent decades, the application of simulation education has increased dramatically worldwide.

Simulation involves a range of types and methods, from low-fidelity simulation to high-fidelity simulation (HFS) (Kim et al., 2016). There has been an increasing utilization of HFS in nursing education since the 1990s (Au et al., 2016). HFS has become the most significant pedagogic strategy for narrowing the “know” versus “do” gap (Hung et al., 2016). HFS refers to the use of a computer-controlled, full-size manikin to demonstrate realistic clinical manifestations and clinical scenarios (Au et al., 2016). Emerging research indicates that HFS helps nursing students develop effective non-technical skills and practice rare emergency situations and presents a variety of authentic, life-threatening situations without compromising the patient’s well-being through the application of realistic clinical scenarios (Hung et al., 2016; Warren et al., 2016).

High-fidelity simulation is a resource-consuming learning strategy (Warren et al., 2016). As an important and necessary part of the teaching process, learning evaluations can help teachers assess students’ learning and performance and further improve and refine their teaching resources (Chen et al., 2015). Although a body of research indicates that HFS might positively impact psychomotor skills, self-efficacy, satisfaction, and critical thinking (Chen et al., 2015; Warren et al., 2016), the evidence regarding HFS effectiveness in student learning is still inconsistent and mixed (Chen et al., 2015; Sundler et al., 2015; Cant and Cooper, 2017). Thus, with regard to the usefulness of HFS, many nurse educators continue to struggle with how to evaluate the effectiveness of simulations.

In China, the integration of HFS in nursing curricula has been at the initial stage in recent years, and approximately 21 universities with undergraduate nursing programs have adopted this type of simulation in nursing education (Gu et al., 2018). However, no practice guidelines for simulation teaching have been published by the Chinese Ministry of Education or other associated institutions, which is partly due to a lack of valid tools or methods for evaluating simulation teaching (Gu et al., 2018). At present, the main evaluation methods for assessing HFS’s effectiveness focus on subjective and objective measures. The objective measures include knowledge tests or skill measures, such as checklists or objective structured clinical examinations (OSCEs), but OSCEs are time-consuming and very expensive (Hung et al., 2016). The most common evaluation tool is self-reported measures (e.g., a subjective appraisal or questionnaire). Several instruments have been developed to evaluate the effectiveness of HFS teaching on student learning, but there are some limitations (Chen et al., 2015; Hung et al., 2016). First, the existing instruments emphasize specific domains or tasks related to simulation learning outcomes, such as clinical ability or confidence, rather than the overall learning effectiveness associated with HFS. Second, there is insufficient evidence for a theoretical framework, and psychometric factors and small sample size are concerns.

The framework of the simulation model is commonly used to guide the process of designing, implementing and evaluating simulations in nursing facilities (Jeffries and Rogers, 2007). Jeffries (2007) noted that to maximize effective use of HFS, it is necessary to evaluate students’ learning experiences and outcomes over the phases of HFS (Jeffries and Rogers, 2007). To our knowledge, the Simulation Learning Effectiveness Inventory (SLEI) is one of the few instruments based on a simulation model that can be applied to evaluate the overall learning effectiveness of the process of HFS in nursing students, and the SLEI was developed and validated in Taiwan (Chen et al., 2015). The SLEI is a 32-item, self-administered questionnaire consisting of three subscales. The first component includes preparation, course arrangement, and equipment resources. Students are asked, during the process of preparing to participate in a simulated scenario, to assess the appropriateness for their learning of the objectives and course activities involved in HFS and the feasibility of environmental and equipment orientations. The second component refers to the HFS process debrief, which is described as a reciprocal and collaborative learning experience in the simulation process. The third component concerns students’ learning outcomes, including problem solving, collaboration, clinical ability, and confidence. For example, the clinical ability and confidence factors respectively assess the extent to which simulation teaching helps students improve their ability and confidence related to taking care of patients with various clinical problems (Chen et al., 2015). It seems that the SLEI is a valid and reliable tool for understanding students’ views on different aspects of learning from the perspective of students’ learning needs so that nursing teachers can better focus resources on HFS teaching and learning (Chen et al., 2015).

The development of simulations for nursing education occurred a few years later in mainland China compared to Taiwan. In recent years, with the rapid improvement in nursing simulation education in mainland China, the cultural and social differences between these two regions have weakened. However, barriers are inevitable when directly applying the HFS method with mainland Chinese nursing students due to linguistic differences between the two regions (Wang et al., 2013). Furthermore, although the traditional Chinese version of the SLEI was found to be valid and reliable, the psychometric characteristics of the scale were not examined adequately; for example, the test–retest reliability was unknown, and the findings implied item redundancy (Chen et al., 2015). Thus, further item-level analysis may offer a promising solution. The psychometric analysis of the SLEI by Chen focused on the classical theory test (CTT), which is based on a raw score across all items (Huang et al., 2017).

Differing from the CTT, IRT analysis provides information about the relation between an individual’s item response (e.g., item discrimination and item difficulty) and underlying latent traits (Hambleton et al., 1991). In recent years, IRT analysis has been increasingly used in nursing education. As an item-level analysis method, it seems to provide more valuable, refined, and comprehensive psychometric information than the CCT (Nicholson et al., 2013). As a so-called “rating scale parametrization,” the graded response model (GRM) estimates one threshold parameter between consecutive response categories for each item, thus achieving a saturated description of their locations in the sense that no constraints are imposed on them (Lubbe and Schuster, 2019). It is widely used for Likert-type data from unidimensional measures in IRT analysis and for item reduction of a scale (Samejima, 2016). For example, Zhao et al. (2018) applied the GRM to the item calibration of the learning instrument (Zhao et al., 2018). Therefore, the objective of this study was to further test the psychometric properties of the SLEI using simplified Chinese characters (SLEI-SC) in mainland China by combining both the CTT and IRT approaches.

## Materials and Methods

### Participants

From May 2017 to July 2018, a total of 533 third-year undergraduate nursing students from the 4-year nursing bachelor’s program at a medical university located in southern China were recruited by a non-probability, convenience purposive sampling method. Students who were willing to participate were invited to respond to the questionnaire. To evaluate test–retest reliability, after 2 weeks, 20% of the participants (*n* = 107) were randomly selected by computer randomized numbers and asked to again complete the instruments. Finally, a total of 533 (response rate 96.1%) undergraduate nursing students completed the questionnaires. Eighty-seven percent of students were female, with an average age of 21.44 years (*SD* = 0.874). The mean frequency of role-playing activities they participated in was 12 (*SD* = 4.05).

### Outcome Measure

The demographic questionnaire and the SLEI were measured pre- and post-course. The SLEI is a self-administered questionnaire consisting of 32 items and seven factors measuring course arrangement (3 items), equipment resources (4 items), debriefing (4 items), clinical abilities (5 items), problem solving (7 items), confidence (5 items), and collaboration (4 items). The seven factors were further divided into three subscales: preparation, process, and outcome. A five-point Likert-scale (1 = “strongly disagree” and 5 = “strongly agree”) was used, with higher scores indicating increased learning effectiveness. The reliability and validity of the scale in Taiwanese nursing students was found to be satisfactory (Chen et al., 2015).

In the current study, the SLEI was converted to a simplified Chinese version (SLEI-SC). In order to ensure it could be easily understood by Mandarin-speaking nursing students, minor modifications were made to simplify a few expressions. For example, the respondent would be likely to better understand “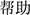,” “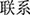,” and “
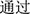
” than “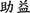,” “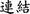,” and “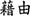.” Then, Shiah-Lian Chen, the developer of the scale, verified the accuracy of the translation. Before the formal survey, a pilot test was performed to examine the clarity and relevance of the item descriptions for the SLEI-SC. Fifty nursing students were asked whether they had any difficulties understanding and answering the items on the questionnaire. All items were understood perfectly and completed without difficulty.

After completing the HFS course, all nursing students received a final course grade, 70% of which represented the regular grade (e.g., attendance, experience reports and other behaviors in class), and 30% of which was the final examination grade (e.g., theoretical case analysis and technical operation score).

### Statistical Analysis

Data analysis was conducted using Mplus 6.1 and SPSS 17.0 software (IBM, Chicago, IL, United States), and *p* < 0.05 was considered significant.

Factorial validity was evaluated by exploratory factor analysis (EFA) and confirmatory factor analysis (CFA). The total sample was randomly split into two sets by propensity score matching. For the EFA, the first set (*n* = 266) was used, and principal axis factor analysis with oblique rotation criterion was applied (Streiner et al., 2015). A scree plot with eigenvalues > 1 and factor loadings > 0.4 was extracted and analyzed.

The IRT analysis used the total sample (*n* = 533) with the marginal maximum likelihood method. Before parameter estimation, the unidimensionality and local item independence of the scale were checked by factor analysis (Hambleton et al., 1991). According to the criteria proposed by Hambleton, if the first factor accounts for more than 20% of the variance, and if the eigenvalue of the first factor divided by the second factor is greater than 3, unidimensionality and local item independence can be assumed (Hambleton et al., 1991).

Then, the appropriate IRT model was chosen according to the model-data fit. According to Drasgow et al. (1995), if the average adjusted chi-square index, which is adjusted chi-square/degrees of freedom, for the scale is equal to a value of 3 or less, the model exhibits a good fit and indicates support for the use of GRM (Drasgow et al., 1995). In this study, the Samejima GRM was used. The estimates of item functioning were described by a discrimination parameter (*a*), difficulty parameters (β_1_, β_2_, β_3_, β_4_), and item characteristic curves. Test information functions (TIFs) were produced from the sum of each item information curve in each subscale and depicted at which levels of θ the scale most precisely and reliably gathers information (Baker, 2001). Items with the largest *a* discriminate the most precisely, while those with largest β*_ik_* correspond to the concept that a step up to the next response option is more difficult and represents lower levels of learning effectiveness. The item characteristic curves provide information about students’ use of the response categories (Samejima, 2016; Palmgren et al., 2018).

The items were refined and reduced according to the findings of the EFA and IRT analysis. The criteria for the parameter estimate of the GRM were as follows: (1) *a* < 0.38, (2) a range of β*_ik_* outside [−3,3] or disordinal or reversal, and (3) the average item information function was less than 0.5. Additionally, (4) the presentation of item characteristic curves was considered; that is, curves 1 and 5 were monotonically distributed, and curves 2, 3, and 4 were normally distributed. If the parameter estimate for the SLEI-SC item did not meet two of the above criteria, it could be considered for deletion (Hambleton et al., 1991).

Following the EFA and IRT analysis, a CFA was performed by using the maximum likelihood method on the second set (*n* = 267). According to the findings of the EFA and IRT, we identified three models, including a six-factor model, a 6 first-order 3 second-order model, and a 6 first-order 1 second-order model. In addition, we compared these three models with the original seven-factor model for the SLEI (Chen et al., 2015). The following absolute and relative indices were employed: normed χ^2^ (χ^2^*/df*) between 1.0 and 3.0 (*p* > 0.05), root mean square error of approximation (RMSEA < 0.08), comparative fit index (CFI), Tucker–Lewis index (TLI), normed fit index (NFI), goodness-of-fit index (GFI), and the Akaike information criterion (AIC). The values for the CFI, TLI, NFI, and GFI should be greater than 0.9, while the value for the AIC should be smaller to obtain the most parsimonious model fit (Zhao, 2014).

To obtain evidence of the reliability of the SLEI-SC, the CTT approach, Cronbach’s α, mean inter-item correlations (MIIC), and test–retest reliability using the intraclass correlation coefficient (ICC) were employed (Zhao, 2014). The marginal reliability evidence from the IRT analysis was also evaluated.

Furthermore, an independent-samples *t* test was used to assess responsiveness to change in the scale by comparing the total pre- and post-course scores, and the effect size was measured by Cohen’s *d*. The concurrent validity of the SLEI-SC was assessed by Pearson correlation analysis; that is, the study examined the relationships between final course grade for the HFS course and total score on the SLEI-SC.

## Results

### Item Refinement for the SLEI-SC

The results of the EFA indicated that three items exhibited cross-loadings: I11, I16, and I22. In the parameter estimate findings of the GRM, nine items did not meet the criteria for the parameter estimate of the GRM: I2, I4, I6–8, I10, I21–22, and I28. After combining the results from both analyses, one item (I22) was removed.

### Factorial Structure of the SLEI-SC

The results of the EFA, excluding I22, are shown in Table 1. Six factors were extracted with eigenvalues of 4.35 to 2.37, and these together explained 60.84% of the overall variance. The factor loadings for all items were between 0.421 and 0.766 (*p* < 0.01).

**TABLE 1:** Factor structure of the SLEI-SC.

Items	Factor					
	
	1	2	3	4	5	6
**Factor 1: Problem-solving and collaboration**						
(I29) Situational simulation practice enabled me to understand the role that I should play in an interaction with a medical team.	0.652					
(I30) During the interaction in the situational simulation, I was willing to share workload with other team members.	0.637					
(I31) I could discuss patient needs with the medical team by using effective communication skills.	0.629					
(I26) In participating in a situational discussion, I identified solutions to problems by understanding argument to topics.	0.572					
(I27) Simulation courses promoted my problem-solving skills in confronting patient problems.	0.550					
(I28) Situational simulation practice provided opportunities to practice communicating and cooperating with other members in my team.	0.529					
(I25) In participating in simulation learning, I approached solutions to problems through data search.	0.517					
(I23) In participating in simulation learning, I approached new concepts or ideas through observation.	0.497					
(I22) Simulation learning enabled me to identify problems in clinical care that I have not noticed before.	0.443					
(I24) Simulation learning enabled me to learn previously unfamiliar learning methods.	0.427					
**Factor 2: Confidence**						
(I21) Simulation learning contributed to my confidence in future patient care.		0.766				
(I19) Simulation learning boosted my confidence in handling future clinical problems.		0.718				
(I18) Situational simulation practice boosted my confidence in my clinical skills.		0.713				
(I20) Simulation learning alleviated my anxiety/fear of confronting future clinical patient problems.		0.686				
(I17) Situational simulation practice encouraged me to confront future clinical challenges.		0.630				
**Factor 3: Clinical ability**						
(I13) Situational learning promoted my ability to care for patients.			0.605			
(I14) Situational learning contributed to my mastering the processes of clinical care.			0.566			
(I12) Situational learning enhanced my understanding of patient problems.			0.544			
(I15) Situational learning enabled me to acquire useful knowledge about clinical practices.			0.503			
(I16) The contents of situational learning corresponded to my previous learning experience.			0.421			
**Factor 4: Debrief**						
(I9) The feedback provided by the teacher was immediate and promoted my learning outcome.				0.754		
(I8) The teacher provided appropriate positive feedback according to the learning situation of students.				0.654		
(I10) Discussion with the teacher after class assisted my achieving the learning goals.				0.593		
(I11) Feedback and discussion of the simulation assisted me in correcting my mistakes and promoting my learning.				0.444		
**Factor 5: Resource**						
(I6) Using the environment and equipment for situational exercises was convenient.					0.633	
(I4)The equipment and resources for situational exercises were sufficient.					0.600	
(I7) If I experienced problems or difficulty using the equipment, help was always available.					0.579	
(I5) The equipment and resources for situational exercises contributed to my learning.					0.493	
**Factor 6: Course**						
(I3) The activities in this course assisted my achieving the learning goals.						0.566
(I1)The course contents were arranged adequately in terms of sequential order and depth, facilitating my learning.						0.553
(I2) I understand the objective and evaluation requirements of this course.						0.428

*The KMO test (0.932), Bartlett’s test was significant (*df* = 496, *p* = 0.000).*

**FIGURE 1:**
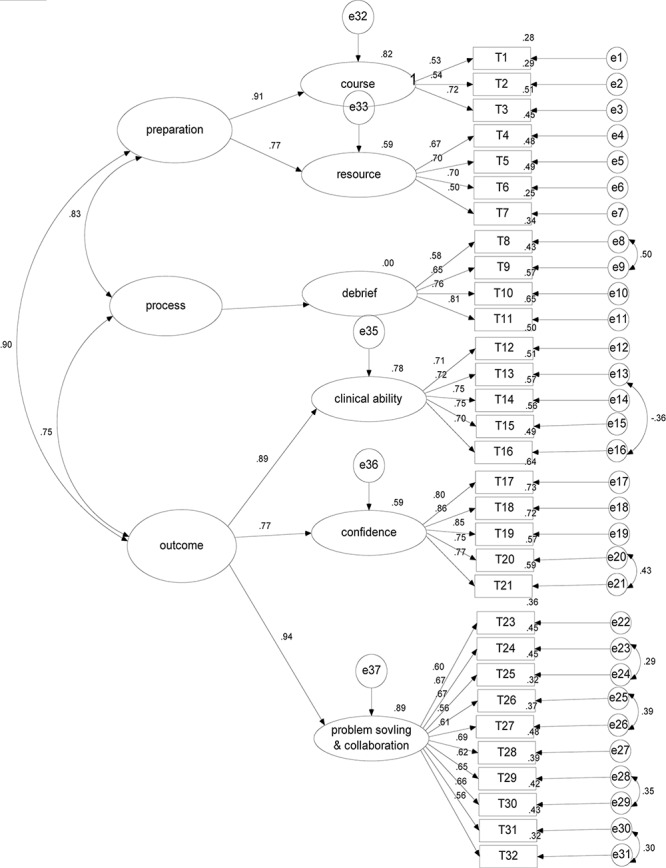
The 6-factor model of the SLEI-SC with standardized estimates (*N* = 267). SLEI-SC indicates the simulation learning effectiveness inventory-a simplified Chinese version.

The six-factor model proposed by the EFA was different from but thematically consistent with the original seven-factor model. In this study, the problem solving and collaboration factors were grouped into one dimension. A series of CFAs were then used to further confirm the factor structure of the SLEI-SC. As shown in Table 2, all the models exhibited satisfactory fit to our data, but the 6 first-order 3 second-order model outperformed the other models (ΔAIC > 10). Therefore, we determined that the 6 first-order 3 second-order structure was the most appropriate (Figure 1). As shown in Figure 1, to improve the model fit, seven pairs of residual correlations were found through the CFA, indicating that they each element of a pair addresses the same facet of the underlying factor; for example, I20 and I21 both measure confidence in confronting a clinical problem.

**TABLE 2:** Model fit indices of different models of the SLEI-SC.

	χ^2^	χ^2^/df*	RMSEA	CFI	GFI	NFI	TLI	AIC
Seven-factor model	754.62	1.76	0.056	0.92	0.86	0.83	0.90	950.43
Six-factor model	723.52	1.75	0.052	0.93	0.86	0.85	0.92	937.76
Six first-order 3 second-order model	721.76	1.72	0.051	0.93	0.88	0.85	0.92	889.52
Six first-order 1 second-order model	755.25	1.79	0.053	0.92	0.86	0.84	0.91	965.25

SLEI-SC, the simulation learning effectiveness inventory—a simplified Chinese version; RMSEA, root mean square error of approximation; CFI, comparative fit index; GFI, goodness-of-fit index; NFI, normed fit index; TLI, Tucker–Lewis index; AIC, Akaike information criterion. *p > 0.05.

### Parameter Estimate of the GRM

The EFA of the three subscales showed that the percentage of variance accounted for by the first factor ranged from 30.75% to 50.12%, and the eigenvalue of the first factor divided by the second factor ranged from 3.14 to 4.26. According to Hambleton’s criteria (Hambleton et al., 1991), these findings indicate the unidimensionality and local item independence of each of the three subscales, which was also confirmed by the CFA (data not shown). According to Drasgow’s criteria (Drasgow et al., 1995), the average adjusted chi-square index was 1.5, supporting the use of the GRM, and we obtained a log likelihood = −3134.17, BIC = 6453.87, and AIC = 6330.42.

The parameter estimates (a, β_1_, β_2_, β_3_, β_4_) for 31 items are presented in Table 3. According to the principles proposed by Hambleton (Hambleton et al., 1991), the slope estimates (*a*) ranged from 0.98 to 2.38, and 96.77% of all items’ *a* values were more than 1, indicating that the majority of the items have the ability to differentiate between levels of a latent trait (learning effectiveness) and are held constant. The difficulty parameter estimates at the first threshold (β_1_) ranged from −5.83 to −2.39, with 41.94% β_1_ outside the criteria of [−3,3] and at the four threshold (β_4_) ranging from 0.93 to 3.11, while no disordinal or reversal β*_ik_* were found. The results indicate that more than half of the items’ β*_ik_* values were appropriate. The maximum value of the item information function ranged from 0.275 (I7) to 1.606 (I15), and 80.65% of all the items’ mean value from the item information function were more than 0.5, indicating that the items in the SLEI-SC offer a broad range and adequate quality of information.

**TABLE 3:** IRT parameter estimates for the SLEI-SC.

Items	Slope	Difficulty				The maximum value of IIF	The mean value of IIF*
				
	α(*SE*)	β_1_(*SE*)	β_2_(*SE*)	β_3_(*SE*)	β_4_(*SE*)		
I1	1.44 (0.15)	−4.49(1.04)	−2.16(0.31)	−0.27(0.11)	2.07 (0.18)	0.567	0.498
I2	1.24 (0.15)	−3.99(0.69)	−2.09(0.31)	0.17 (0.11)	3.02 (0.28)	0.434	0.394
I3	1.70 (0.17)	−3.52(0.70)	−2.23(0.29)	−0.38(0.10)	1.93 (0.15)	0.817	0.662
I4	1.02 (0.13)	−5.43(1.13)	−2.75(0.44)	−0.50(0.17)	2.07 (0.22)	0.295	0.276
I5	1.76 (0.18)	−3.86(0.88)	−2.57(0.39)	−0.54(0.11)	1.52 (0.12)	0.868	0.661
I6	1.33 (0.15)	−3.95(0.72)	−1.85(0.26)	0.09 (0.11)	2.14 (0.19)	0.494	0.453
I7	0.98 (0.13)	−4.51(0.75)	−2.23(0.36)	0.04 (0.14)	3.11 (0.35)	0.275	0.259
I8	1.43 (0.15)	−3.98(0.80)	−2.56(0.34)	−1.05(0.16)	1.23 (0.13)	0.600	0.469
I9	1.75 (0.17)	−3.82(0.97)	−2.42(0.33)	−0.88(0.13)	1.14 (0.10)	0.861	0.659
I10	1.55 (0.16)	−5.58(1.54)	−2.88(1.19)	−1.06(0.18)	1.05 (0.11)	0.653	0.506
I11	1.98 (0.20)	−4.96(1.21)	−2.54(0.75)	−0.65(0.11)	1.07 (0.09)	1.034	0.733
I12	1.98 (0.21)	−5.02(1.44)	−2.09(0.33)	−0.69(0.11)	1.14 (0.10)	1.064	0.727
I13	1.77 (0.18)	−3.39(0.69)	−1.78(0.23)	−0.35(0.09)	1.42 (0.12)	0.870	0.700
I14	2.14 (0.20)	−4.63(1.26)	−2.19(0.48)	−0.68(0.10)	1.06 (0.09)	1.209	0.815
I15	2.36 (0.21)	−2.71(0.48)	−2.18(0.28)	−0.66(0.09)	1.05 (0.08)	1.606	1.017
I16	2.38 (0.22)	−2.86(0.43)	−1.91(0.22)	−0.65(0.09)	1.24 (0.09)	1.596	1.084
I17	2.21 (0.19)	−3.13(0.71)	−1.39(0.15)	−0.14(0.07)	1.40 (0.10)	1.325	0.991
I18	2.14 (0.20)	−2.39(0.36)	−1.28(0.15)	0.05 (0.07)	1.44 (0.11)	1.285	0.947
I19	2.19 (0.19)	−2.97(0.58)	−1.30(0.15)	−0.01(0.07)	1.56 (0.11)	1.294	1.005
I20	1.68 (0.16)	−3.50(0.71)	−1.17(0.16)	0.16 (0.08)	1.77 (0.15)	0.806	0.649
I21	1.93 (0.17)	−2.97(0.52)	−1.21(0.15)	0.11 (0.08)	1.78 (0.13)	1.027	0.843
I22	2.19 (0.21)	−4.89(1.33)	−2.07(0.30)	−0.74(0.11)	0.93 (0.08)	1.289	0.819
I23	2.26 (0.20)	−4.51(0.75)	−1.82(0.28)	−0.25(0.08)	1.36 (0.09)	1.343	0.884
I24	2.31 (0.21)	−3.24(0.88)	−1.76(0.21)	−0.39(0.08)	1.27 (0.09)	1.418	1.039
I25	1.54 (0.16)	−5.83(1.43)	−2.70(0.68)	−0.79(0.14)	1.36 (0.12)	0.636	0.513
I26	1.94 (0.19)	−3.58(0.93)	−2.20(0.28)	−0.45(0.09)	1.37 (0.11)	1.025	0.784
I27	2.26 (0.22)	−4.58(1.11)	−2.04(0.36)	−0.45(0.09)	1.36 (0.10)	1.323	0.888
I28	1.95 (0.18)	−5.10(1.09)	−2.24(0.36)	−0.71(0.12)	1.08 (0.09)	1.020	0.712
I29	2.22 (0.20)	−2.71(0.49)	−1.88(0.23)	−0.67(0.10)	1.13 (0.09)	1.449	0.961
I30	2.30 (0.20)	−2.76(0.49)	−1.85(0.19)	−0.64(0.09)	1.04 (0.09)	1.514	1.014
I31	1.94 (0.18)	−5.10(1.09)	−2.00(0.32)	−0.32(0.09)	1.30 (0.11)	0.999	0.711

IIF, item information function. *The mean item information function of seven categories, that is category −3, −2, −1, 0, 1, 2, 3.

The item characteristic curves and TIFs for each of the three subscales are shown in the Supplementary Appendix. All item characteristic curves were well-shaped; the peak of five curves did not overlap, and curves 2, 3, and 4 were normally distributed. Regarding the TIFs, three subscales of the SLEI-SC gathered information very precisely at θ, ranging from −2.0 to 1.5, indicating that when students estimated learning effectiveness levels ranging from −0.5 to 1.5, −2 to 0.5, −0.5 to −1.5, and 0.5 to 1.5, the scale provided the most precise information with the lowest standard error.

### Reliability

The SLEI-SC achieved a Cronbach’s α of 0.88 (each factor: 0.71–0.90), an MIIC of 0.38 (each factor: 0.43–0.65), and a marginal reliability of 0.96 (each factor: 0.80–0.95). The test–retest reliability for the total scale was good, with an ICC of 0.88.

### Responsiveness

The total score for learning effectiveness post-course was better than the total score pre-course, and the difference was statistically significant [*t* = 2.59, *p* < 0.05, power (1 −β) = 0.95, Cohen’s *d* = 0.60].

### Convergent Validity

The relationship between final course grade and total score on the SLEI-SC was statistically significant (*r* = 0.63, *p* < 0.05).

## Discussion

This paper profiled the psychometric testing of the SLEI-SC through the CTT and IRT approaches. This scale was adapted from the SLEI, which has been previously reported to show consistent reliability and validity with nursing students in Taiwan (Chen et al., 2015). To the best of our knowledge, this is the first (albeit preliminary) study to investigate the learning effectiveness of HFS teaching as perceived by nursing students in mainland China, which provides important information for a further large-scale investigation and serves as a basis for improving students’ learning efficiency through simulation programs.

Measurement of learning effectiveness is an important part of evaluating the implementation of HFS education initiatives because it can help nursing teachers assess student learning and performance and further improve and refine their teaching. According to the Jeffries’ simulation model (Jeffries and Rogers, 2007), education practices, simulation design, and learning outcomes are all favorable factors contributing to simulation teaching and learning. The SLEI-SC was found to be a reliable and valid instrument for measuring these favorable factors in relation to an HFS learning experience. In our study, the findings added evidence regarding the factorial validity of the SLEI-SC. We highlight a 6 first-order 3 second-order model rather than the 7 first-order 3 second-order model employed in the original study. The three second-order components corresponded closely with the components of the original SLEI and the simulation model: preparation, process, and outcome.

These outcomes derived from the SLEI reflect the unique contribution and value of HFS. Unlike in the original scale, factor collaboration and problem-solving ability were combined into one dimension, called “problem solving.” The findings further highlight the phenomenon of growing attention on multidisciplinary collaboration in healthcare practice. When nursing students engage in problem-solving activities, they need to not only think about how to resolve a clinical situation but also know the roles and role expectations of team members and be able to communicate effectively with each other (Hung et al., 2016). Thus, the SLEI-SC may help faculty understand the extent to which simulation teaching improves students’ clinical ability, problem solving, and confidence.

As reported by Chen et al. (2015), the original SLEI exhibited item redundancy, and this item redundancy needed to be reduced for scale parsimony (Chen et al., 2015). In our study, CTT and IRT approaches were combined to refine the items and evaluate the psychometric characteristics of the scale. According to the results of the CTT and IRT approaches, one item (I22), “Simulation learning enabled me to understand the implication of each solution for patient problems,” was deleted. A possible explanation could be the limitations of the HFS strategy with regard to authenticity and complexity; that is, it might be difficult for some students to accept HFS with a manikin as “the patient,” and HFS cannot fully replace the context of real-life healthcare because patients’ concerns and responses are complicated and changeable (Warren et al., 2016). Consequently, the use of HFS could influence and limit learners’ understanding of the implication of solutions to problems.

Using CTT, our data support that the SLEI-SC has satisfactory internal consistency, good temporal stability, and high sensitivity in addition to exhibiting robust evidence of factorial validity. The statistically significant difference between nursing students’ learning effectiveness pre- and post-course highlights the sensitivity characteristics of the SLEI-SC. On the other hand, we used an objective evaluation index, that is, final course grade, to further validate the concurrent validity of the scale. Both of them indicate that the SLEI-SC may not only be useful for evaluating nursing students’ learning effectiveness, and the gap of nursing HFS teaching, but also imply the HFS teaching is effective.

Using IRT, we further confirmed the reliability of the scale and found that the items on the SLEI-SC not only have the ability to discriminate between undergraduate nursing students with high and low levels of learning effectiveness but also perform well over a broad range of nursing undergraduate students (Embretson and Reise, 2013; Samejima, 2016; Palmgren et al., 2018). Regarding the TIF of the scale, when represented graphically, high TIF values are associated with low standard errors of measurement, thus indicating precision (Hambleton et al., 1991). The most precise information provided by the TIFs for the three subscales of the SLEI-SC indicated that the SLEI-SC appears to precisely and reliably measure learning effectiveness among nursing students perceiving low to moderate levels of learning effectiveness.

Robust psychometric characteristics and the practicality of a tool represent an important basis for the accumulation of empirical data in the literature (Cant and Cooper, 2017). Overall, there is sufficient evidence of reliability and validity to support the utilization of the SLEI-SC in Chinese nursing education programs. The SLEI-SC provides nursing teachers a reliable and valid tool for quickly and completely evaluating nursing students’ learning efficacy with regard to the entire HFS curriculum and acknowledges the deficiencies inherent in the HFS teaching process. Thus, it provides direction for nursing teachers to design and revise HFS curriculum. For example, through the debriefing process, students and facilitators may have the opportunity to re-examine the assimilability and transferability of the simulation experience. In other words, the process helps students move toward assimilation and accommodation of learning to enable the transfer of learning to new situations. Based on nursing students’ views on debriefing, nurse educators could continuously explore the development of debriefing methodologies, which are effective in nursing students’ structured learning environments (Neill and Wotton, 2011). Furthermore, the SLEI-SC provides a sensitive evaluation tool for nursing scholars to compare the learning effectiveness of different teaching strategies. The ultimate goal is to assist nursing students in transferring learning to clinical practice in the unpredictable and unstable healthcare environment (Lestander et al., 2016).

## Study Limitations

This study has some limitations that should be considered. First, participants were recruited based on convenience sampling of undergraduate nursing students in one region of mainland China, potentially limiting the generalizability of the findings to other Chinese−speaking areas. Second, we need additional evidence of construct validity; for example, differential item function analysis is an important next step for examining the discriminate validity of the scale. In this study, only one item was deleted, and seven pairs of residual correlations were found in the CFA results, indicating that the SLEI-SC must be further refined with a larger representative sample to produce more stable parameter estimates and robust results.

## Conclusion

The psychometric properties presented here support the use of the 31-item SLEI-SC with 6 subdomains as a measure of learning effectiveness in HFS teaching among Chinese nursing students. This instrument is useful, as it provides a means for teachers to better understand the learning outcomes of nursing students from the perspectives of the preparation, process, and outcomes associated with HFS. Furthermore, it can facilitate the customization and optimization of HFS program use with nursing students.

## Data Availability Statement

The datasets analyzed in this manuscript are not publicly available. Requests to access the datasets should be directed to pt860315@163.com.

## Ethics Statement

The study was approved by the ethical committee of Fujian Medical University (No: 2017052).

## Author Contributions

FH contributed to the conception and design of the work. FH and S-LC contributed to the interpretation of data and the drafting of the manuscript. FH and XY contributed to the revision of the manuscript. XY and QZ contributed to the data collection and analysis. YG and AW contributed to the translation of instruments. All authors contributed to approve the final manuscript for publication.

## Conflict of Interest

The authors declare that the research was conducted in the absence of any commercial or financial relationships that could be construed as a potential conflict of interest.
