# Supplementary material for: Psychometric Testing of the Chinese Simple Version of the Simulation Learning Effectiveness Inventory: Classical Theory Test and Item Response Theory
Source: Front Psychol. 2020 Feb 19;11:32. doi: 10.3389/fpsyg.2020.00032 (PMC7042430; doi:10.3389/fpsyg.2020.00032)
Supplement: Supplementary file 1 [file Data_Sheet_1.docx]

**Appendix**

Figure 1. The test infromation curve of the preparation subscale

Figure 2. The test infromation curve of the process subscale

Figure 3. The test infromation curve of the outcome subscale

Figure 4. The test characteristic curves of the items
